# Interacting Cannabinoid and Opioid Receptors in the Nucleus Accumbens Core Control Adolescent Social Play

**DOI:** 10.3389/fnbeh.2016.00211

**Published:** 2016-11-16

**Authors:** Antonia Manduca, Olivier Lassalle, Marja Sepers, Patrizia Campolongo, Vincenzo Cuomo, Giovanni Marsicano, Brigitte Kieffer, Louk J. M. J Vanderschuren, Viviana Trezza, Olivier J. J. Manzoni

**Affiliations:** ^1^Institut National De La Santé Et De La Recherche Médicale U901Marseille, France; ^2^Université de la Méditerranée UMR S901 Aix-Marseille 2Marseille, France; ^3^INMEDMarseille, France; ^4^Department of Physiology and Pharmacology, Sapienza University of RomeRome, Italy; ^5^NeuroCentre Magendie, Endocannabinoids and Neuroadaptation, Institut National De La Santé Et De La Recherche Médicale U862Bordeaux, France; ^6^NeuroCentre Magendie U862, University of BordeauxBordeaux, France; ^7^Institut de Génétique et de Biologie Moléculaire et Cellulaire, Centre National de la Recherche Scientifique/Institut National de la Santé et de la Recherche Médicale/Université de StrasbourgIllkirch, France; ^8^Division of Behavioural Neuroscience, Department of Animals in Science and Society, Faculty of Veterinary Medicine, Utrecht UniversityUtrecht, Netherlands; ^9^Section of Biomedical Sciences and Technologies, Department of Science, University Roma TreRome, Italy

**Keywords:** accumbens, opioid, cannabinoid, social play

## Abstract

Social play behavior is a highly rewarding, developmentally important form of social interaction in young mammals. However, its neurobiological underpinnings remain incompletely understood. Previous work has suggested that opioid and endocannabinoid neurotransmission interact in the modulation of social play. Therefore, we combined behavioral, pharmacological, electrophysiological, and genetic approaches to elucidate the role of the endocannabinoid 2-arachidonoylglycerol (2-AG) in social play, and how cannabinoid and opioid neurotransmission interact to control social behavior in adolescent rodents. Systemic administration of the 2-AG hydrolysis inhibitor JZL184 or the opioid receptor agonist morphine increased social play behavior in adolescent rats. These effects were blocked by systemic pretreatment with either CB1 cannabinoid receptor (CB1R) or mu-opioid receptor (MOR) antagonists. The social play-enhancing effects of systemic morphine or JZL184 treatment were also prevented by direct infusion of the CB1R antagonist SR141716 and the MOR antagonist naloxone into the nucleus accumbens core (NAcC). Searching for synaptic correlates of these effects in adolescent NAcC excitatory synapses, we observed that CB1R antagonism blocked the effect of the MOR agonist DAMGO and, conversely, that naloxone reduced the effect of a cannabinoid agonist. These results were recapitulated in mice, and completely abolished in CB1R and MOR knockout mice, suggesting that the functional interaction between CB1R and MOR in the NAcC in the modulation of social behavior is widespread in rodents. The data shed new light on the mechanism by which endocannabinoid lipids and opioid peptides interact to orchestrate rodent socioemotional behaviors.

## Introduction

Social play behavior is a highly vigorous form of social interaction, which is abundant in young mammals, including humans (Panksepp et al., 1984; Pellis and Pellis, 2009). In terms of structure, social play behavior contains elements of sexual, aggressive, and affiliative behavior, but in an out-of-context and modified, often exaggerated fashion (Panksepp et al., 1984; Vanderschuren et al., 1997; Pellis and Pellis, 2009). Social play is thought to facilitate neurobehavioral development (Pellis and Pellis, 2009; Vanderschuren and Trezza, 2014), and abnormal social play is observed in child and adolescent psychiatric disorders (Alessandri, 1992; Jordan, 2003).

Social play has a strong rewarding value (Calcagnetti and Schechter, 1992; Trezza et al., 2011b; Achterberg et al., 2016). It can be used as an incentive for place conditioning, operant conditioning and maze learning in laboratory animals (for a review see Panksepp et al., 1987; Trezza et al., 2011a), and social play is modulated by neurotransmitters implicated in reward and motivation, such as endogenous opioids, endocannabinoids (eCBs), and dopamine (Trezza and Vanderschuren, 2008b; Trezza et al., 2010; Achterberg et al., 2016; Manduca et al., 2016; Vanderschuren et al., 2016). The nucleus accumbens (NAc) has been identified as an anatomical substrate for motivation and reward (Everitt and Robbins, 2005; Berridge and Kringelbach, 2015; Floresco, 2015). Indeed, the NAc has been shown to be involved in social play behavior in rats (Vanderschuren et al., 1995b; Gordon et al., 2002; Trezza et al., 2011b, 2012; van Kerkhof et al., 2013, 2014; Manduca et al., 2016). Notably, mu-opioid receptor (MOR) activation in the NAc stimulates social play in adolescent rats (Trezza et al., 2011b). Likewise, intra-NAc infusion of the dopamine releaser/reuptake inhibitor amphetamine increases social play behavior, and the social play-enhancing effects of increased opioid and eCB neurotransmission depend on stimulation of dopamine receptors in the NAc (Manduca et al., 2016). Interestingly, with regard to the modulation of social play by local opioid and dopamine signaling, there does not seem to be a major difference between the NAc core (NAcC) and shell subregions (Trezza et al., 2011b; Manduca et al., 2016).

In the CNS, two principal eCBs are thought to be responsible for the neuromodulatory functions of CB1 cannabinoid receptors (CB1R): anandamide and 2-arachidonoylglycerol (2-AG; Katona and Freund, 2012). Both molecules are synthesized on-demand to regulate synaptic transmission (Castillo et al., 2012). Aberrant eCB signaling in the NAc has been implicated in several emotional disorders including anxiety, depression and addiction (Kasanetz et al., 2010; Lafourcade et al., 2011; Jung et al., 2012). Anandamide stimulates social play via CB1R located in the basolateral amygdala and the NAc, whereby the former structure appears to play a more prominent role (Trezza et al., 2012). 2-AG is known to regulate emotional behaviors in rodents (Campolongo and Trezza, 2012; Mulvihill and Nomura, 2013) and it mediates synaptic plasticity in limbic brain areas, such as the NAc, the amygdala and the prefrontal cortex (Puente et al., 2011; Katona and Freund, 2012).

CB1R and opioid receptors (OR) exhibit overlapping mechanisms and they are likely interact to modulate multiple behaviors (Navarro et al., 2001; Fattore et al., 2005; Mackie, 2005; Hudson et al., 2010; Pertwee, 2010; Befort, 2015) starting at early developmental stages (Ellgren et al., 2007, 2008; Biscaia et al., 2008; Naudon et al., 2013). Indeed, opioid and eCB neurotransmission have been shown to interact in the modulation of social play (Trezza and Vanderschuren, 2008a, 2009), but it is not known whether this interaction occurs at the level of the NAc. Moreover, the contribution of the endocannabinoid, 2-AG, to social play remains unknown.

Here we combined behavioral, pharmacological, electrophysiological and genetic methods to address these questions. To test the role of 2-AG in social behavior in adolescent rats and mice, we relied on the well-characterized 2-AG hydrolysis inhibitor JZL184, which prolongs the effects of locally produced 2-AG (Long et al., 2009; Seillier et al., 2014; Morena et al., 2015). We hypothesized that MORs and CB1Rs interact in the modulation of social behavior in young rats and mice.

## Materials and methods

### Animals

Rats and mice arrived in our animal facility at 3 weeks of age and they were housed in groups of five under controlled conditions (i.e., temperature 21 ± 1°C, 60 ± 10% relative humidity and 12 h light/dark cycles). Food and water were available *ad libitum*. All procedures were performed in agreement with the European Communities Council Directive (2010/63/EU) for the Care and Use of Laboratory Animals. For behavioral experiments, male Wistar rats and male C57Bl6/J mice (age 4–6 weeks) were used. For electrophysiological experiments, male Wistar rats (age 4–6 weeks) were used for all experiments unless otherwise stated. Female CB1R null mice (CB1R^−/−^) on a C57Bl6/J genetic background (Sanchis-Segura et al., 2004; age 5–10 weeks) were used to confirm cannabinoid dependent action of DAMGO, with wild type (WT) littermates used as control group (CB1R^+/+^). Female and male MOR receptor null mice (MOR^−/−^) on a 50% C57Bl6/J and 50% 129SvPAS genetic background (Matthes et al., 1996; age 4–7 weeks) were used to test MOR dependent action of CP55940, with WT littermates as control group (MOR^+/+^).

### Behavioral experiments

#### Surgical procedures

The surgical procedures were based on our previous experiments (Trezza et al., 2011b, 2012; Manduca et al., 2016). At 4 weeks of age, rats were anesthetized with sodium pentobarbital (35 mg/kg, i.p.; Sigma Aldrich, Italy) and positioned into a stereotaxic frame (David Kopf Instruments, USA). Guide cannulae (24-gauge; Cooper's Needleworks, UK) were implanted bilaterally, aimed 1.0 mm above the NAcC (coordinates: AP + 1.5 mm; ML ± 1.9; DV − 6.5 mm (Trezza et al., 2011b, 2012). Cannulae were secured with stainless steel screws and dental acrylic; 29-gauge wire stylets (Cooper's Needleworks, UK) were inserted into the guide cannulas to maintain patency. After surgery, the rats were individually housed and allowed to recover for 4 days. On the fifth day, they were re-housed in groups of five with their original cage mates. Behavioral testing began 1 week after surgery.

#### Drugs and infusion procedures

The monoacylglycerol lipase (MAGL) inhibitor JZL184 [4-[Bis(1,3-benzodioxol-5-yl)hydroxymethyl]-1-piperidinecarboxylic acid 4-nitrophenyl ester] and the CB1 receptor antagonist/inverse agonist SR141716 [N-(Piperidin-1-yl)-5-(4-chlorophenyl)-1-(2,4-dichlorophenyl)-4-methyl-1H-pyrazole-3-carboxamide hydrochloride; National Institute of Mental Health's (NIMH) Chemical Synthesis and Drug Supply Program, USA] were dissolved in 5% Tween 80/5% polyethylene glycol/saline. The opioid receptor agonist morphine (SALARS, Italy), the opioid receptor antagonist naloxone hydrochloride (Sigma Aldrich, Italy) and the selective μ-opioid receptor antagonist CTAP (D-Phe-Cys-Tyr-D-Trp-Arg-Thr-Pen-Thr-NH2; Sigma Aldrich, Italy) were dissolved in saline. JZL184 (1–5 mg/kg) and morphine (1 mg/kg) were given intraperitoneally (i.p.) and subcutaneously (s.c.) 2 and 1 h, respectively, before testing. For systemic drug treatment experiments, SR141716 (0.1 mg/kg, i.p.) and naloxone hydrochloride (1 mg/kg, s.c.) were injected 30 min and 1 h before JZL184 and morphine, respectively. For intracranial drug treatment experiments, SR141716 (3 μg/0.3 μl), naloxone hydrochloride (0.5 μg/0.3 μl) and CTAP (0.3 μg/0.3 μl) were infused into the NAcC immediately before testing. Dose ranges were selected based on our previous studies (Trezza et al., 2011b, 2012) and pilot experiments (data not shown). Bilateral infusions of drugs or an equivalent volume of the corresponding vehicle were made as described in our previous studies (Trezza et al., 2011b, 2012; Manduca et al., 2016). After infusions, animals were left in a holding cage for 5 min before testing. At the end of experiments, injection sites were confirmed histologically according to the atlas of rat brain (Paxinos and Watson, 2007) as previously described (Trezza et al., 2011b, 2012). Only pairs in which both animals had bilateral needle tracks terminating into the NAcC were included in the final analysis. For histological assessment of representative experiments see Supplementary Figure 1.

#### Social behavior in adolescent rats and mice

The experiments were performed in a sound attenuated chamber under dim light conditions. The testing arena consisted of a Plexiglas cage measuring 40 × 40 × 60 cm^3^ (l × w × h) with 2 cm of wood shavings covering the floor. Social behavior was assessed as previously described (Terranova and Laviola, 2005; Trezza et al., 2011b, 2012; Janecka et al., 2015).

**Figure 1:**
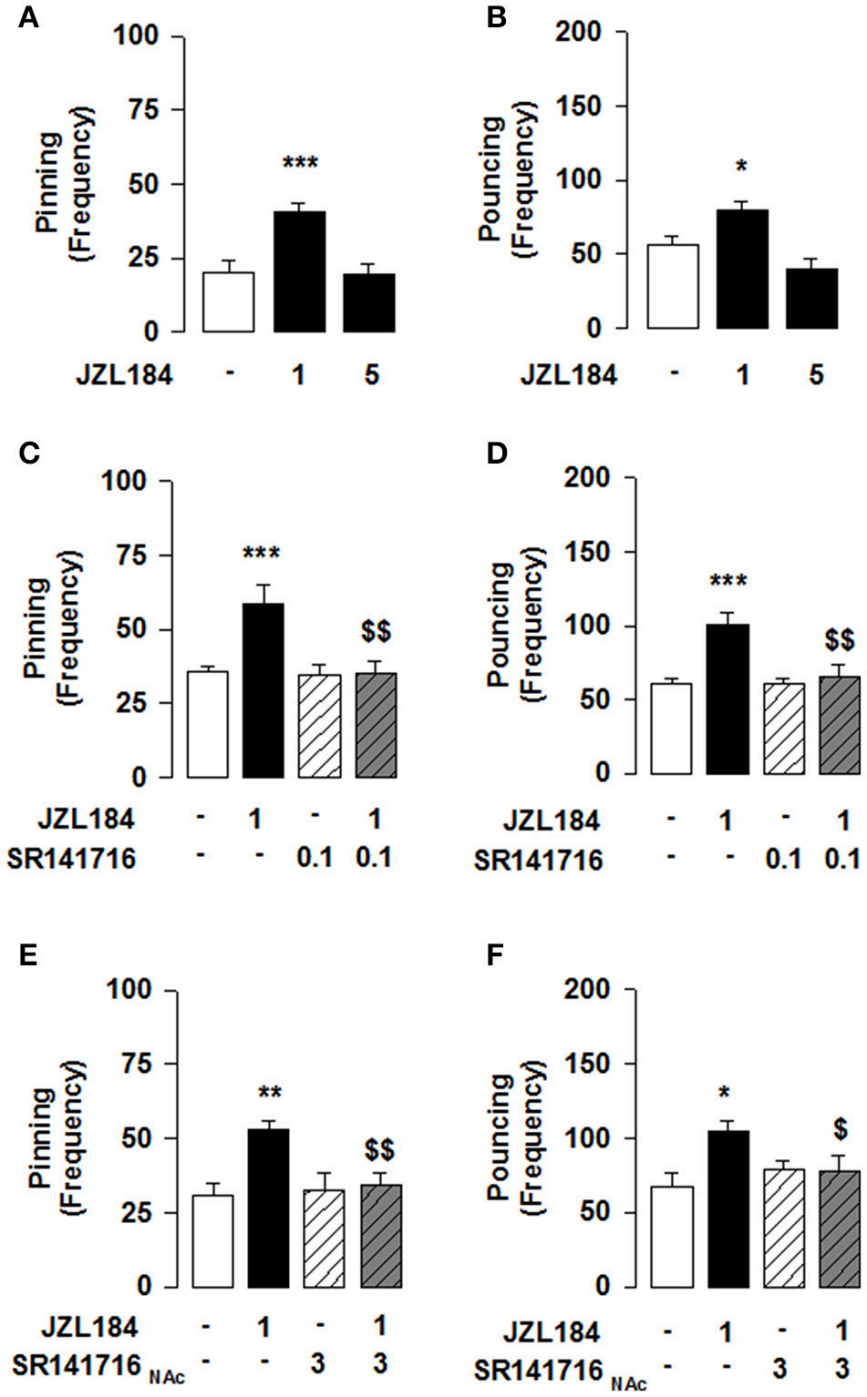
**2-AG elevation stimulates social play via CB1R in the NAcC in adolescent rats**. JZL184 (1 mg/kg, i.p.) enhanced pinning **(A)** and pouncing **(B)** frequency. Systemic pretreatment with the CB1R antagonist SR141716 (0.1 mg/kg, i.p.) blocked the effects of JZL184 on pinning **(C)** and pouncing **(D)**. Intra-NAcC infusion of SR141716 (3 μg/0.3 μl) antagonized the play-enhancing effects of JZL184 (1 mg/kg, i.p.) **(E,F)**. Data represent mean ± S.E.M. frequency of pinning and pouncing. ^*^p < 0.05, ^**^p < 0.01, ^***^p < 0.001 vs. vehicle; ^*$*^*p* < 0.05, ^*$$*^*p* < 0.01 vs. vehicle/JZL184 (Student-Newman-Keuls *post-hoc* test). *N* = 7–9 per treatment group.

**Figure 2:**
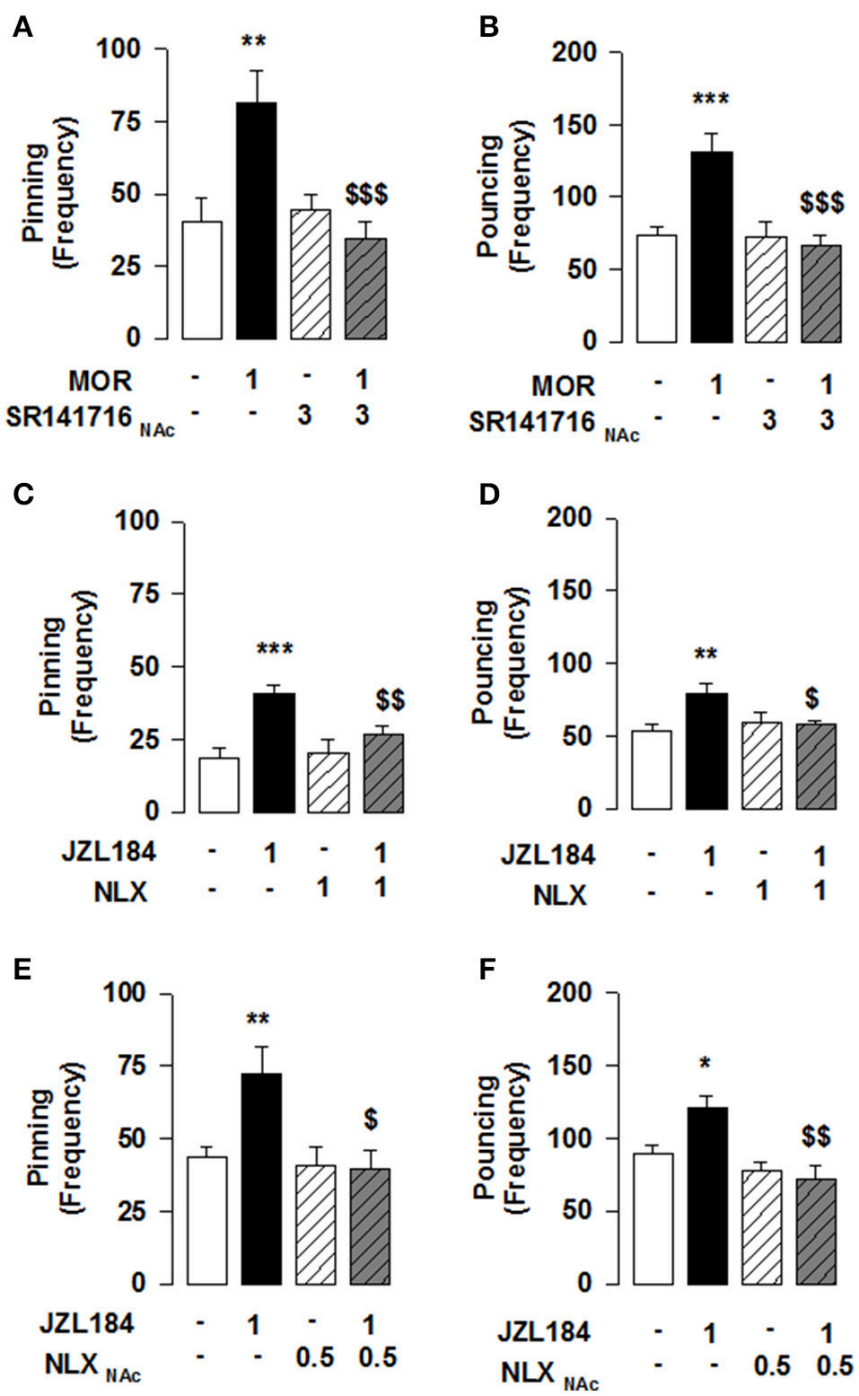
**MOR activation in the NAcC is necessary for the 2-AG induced stimulation of social play in adolescent rats**. Play-enhancing effects of morphine (1 mg/kg, s.c.) were prevented by intra-NAcC infusion of SR141716 (3 μg/0.3 μl) **(A,B)**. Systemic pretreatment with the OR antagonist naloxone (NLX: 1 mg/kg, s.c.) blocked the effects of JZL184 (1 mg/kg, i.p.) on pinning **(C)** and pouncing **(D)**. Intra-NAcC infusion of NLX (0.5 μg/0.3 μl) antagonized the play-enhancing effects of JZL184 (1 mg/kg, i.p.) **(E,F)**. Data represent mean ± S.E.M. frequency of pinning and pouncing. ^*^p < 0.05; ^**^p < 0.01, ^***^p < 0.001 vs. vehicle; ^*$*^*p* < 0.05, ^*$$*^*p* < 0.01, ^*$$$*^*p* < 0.001 vs. vehicle/JZL184 and vehicle/morphine (Student-Newman-Keuls *post-hoc* test). *N* = 6–12 per treatment group.

To evaluate (1) the effects of systemic administration of JZL184 on social play behavior (Figures 1A,B, 4A,B) and (2) whether the effects of JZL184 were mediated by CB1R (Figures 1C,D, **4C,D**) and/or OR activation (Figures 2C,D, **4E,F**), adolescent rats and mice (that had not undergone stereotaxic surgery) were individually habituated to the test cage for 10 min on each of the 2 days before testing. Before testing, adolescent rats were socially isolated for 3.5 h to enhance their social motivation and thus facilitate the expression of social play behavior during testing (Niesink and Van Ree, 1989; Vanderschuren et al., 1995a, 2008). The test consisted of placing the animal together with a similarly treated partner into the test cage for 15 min (rats) or 10 min (mice). To determine whether the increase in social play induced by JZL184 depends upon activation of CB1R (Figures 1E,F) and/or MOR (Figures 2E,F) in the NAcC, rats equipped with bilateral guide cannulae were habituated to the experimental procedures on 2 consecutive days, as previously described (Trezza et al., 2011b; van Kerkhof et al., 2013; Manduca et al., 2016). On the first habituation day, they were individually placed into the test cage for 10 min, and on the second habituation day, they were isolated for 2 h. Pairs of rats were then infused with saline solution and placed into the test cage for 15 min to habituate them to the infusion procedures and determine baseline levels of social play behavior. On the test day, the animals were isolated for 2 h before testing. Pairs of rats were then infused simultaneously with either vehicle or drug solutions and placed into the test cage for 15 min. Behavior was assessed per pair of animals and analyzed by a trained observer who was unaware of the treatment condition, using the Observer 3.0 software (Noldus Information Technology, The Netherlands). Both animals in a test pair received the same treatment. Animals in a test pair did not differ by >10 g in body weight and had no previous common social experience (i.e., they were not cage mates).

In adolescent rats, the following parameters were scored per pair of animals: 1/frequency of pinning: one animal lying with its dorsal surface on the floor with the other animal standing over it. This is the most characteristic posture in social play in rats; it occurs when one animal is solicited to play by its test partner and rotates to its dorsal surface (Panksepp and Beatty, 1980; Pellis and Pellis, 2009; Trezza et al., 2010); 2/frequency of pouncing: one animal is soliciting the other to play, by attempting to nose or rub the nape of its neck (Panksepp and Beatty, 1980; Pellis and Pellis, 2009; Trezza et al., 2010). In addition to these measures, time spent in social exploration (sniffing, licking, or grooming any part of the body of the test partner, including the anogenital area) was also assessed as a measure of general social interest; this may not necessarily be associated with playful social behavior.

In adolescent mice, the total time and total frequency of active social interactions were obtained as the sum of the time and frequency of social sniffing (sniffing any part of the body of the test partner), social grooming (one mouse licks and chews the fur of the conspecific, while placing its forepaws on the back or the neck of the other mouse), following/chasing (walking or running in the direction of the partner which stays where it is or moves away), crawling under/over (one animal crawls underneath or over the partner's body, crossing it transversely from one side to the other) scored per 10 min (Terranova and Laviola, 2005; Janecka et al., 2015) (for the experimental design see Table 1).

**Table 1:** **Behavioral experimental design**.

**Treatment**	**Mechanism of action**	**Animal species used**	**Group treatment**	**Way of injection**
Systemic JZL184 administration (Figures 1A,B, 4A,B)	By enhancing 2-AG levels, systemic administration of JZL184 increased social play behavior in rats and mice	Rats	Control	systemic (i.p.)
			JZL184 1 mg/kg	systemic (i.p.)
			JZL184 5 mg/kg	systemic (i.p.)
		Mice	Control	systemic (i.p.)
			JZL184 4 mg/kg	systemic (i.p.)
			JZL184 8 mg/kg	systemic (i.p.)
			JZL184 16 mg/kg	systemic (i.p.)
Systemic JZL184 + SR141716 administration (Figures 1C,D, 4C,D)	Pretreatment with the CB1R antagonist SR141716 blocked the JZL184 play-enhancing effects on social play behavior in rats and mice	Rats	Control	systemic (i.p.)
			JZL184 1 mg/kg	systemic (i.p.)
			SR141716 0.1 mg/kg	systemic (i.p.)
			SR 0.1 mg/kg + JZL184 1 mg/kg	systemic (i.p.)
		Mice	Control	systemic (i.p.)
			JZL184 8 mg/kg	systemic (i.p.)
			SR141716 3 mg/kg	systemic (i.p.)
			SR 3 mg + JZL184 8 mg/kg	systemic (i.p.)
Intra-NAcC SR141716 after systemic administration of JZL184 (Figures 1E,F)	Intra-NAcC infusion of the CB1R antagonist SR141716 blocked play-enhancing effects of systemic JZL184 treatment in rats	Rats	Control	Intra NAcC
			JZL184 1 mg	systemic (i.p.)
			SR141716 3 μg	Intra NAcC
			SR 3 μg + JZL184 1 mg/kg	Intra NAcC + systemic (i.p.)
Intra-NAcC SR141716 after systemic administration of morphine (Figures 2A,B)	Intra-NAcC infusion of the CB1R antagonist SR141716 blocked the play-enhancing effects of systemic treatment with the opioid receptor agonist morphine in rats	Rats	Control	Intra NAcC
			MOR 1 mg/kg	systemic (s.c.)
			SR141716 3 μg	Intra NAcC
			SR 3 μg + MOR 1 mg/kg	Intra NAcC + systemic (s.c.)
JZL184 + naloxone systemic administration (Figures 2C,D, 4E,F)	Pretreatment with the OR antagonist naloxone blocked the play-enhancing effects of systemic treatment with JZL184 in rats and mice	Rats	Control	systemic (i.p.)
			JZL184 1 mg/kg	systemic (i.p.)
			NLX 1 mg/kg	systemic (s.c.)
			NLX 1 mg/kg + JZL184 1 mg/kg	systemic (s.c.) + systemic (i.p.)
		Mice	Control	systemic (i.p.)
			JZL184 8 mg/kg	systemic (i.p.)
			NLX 1 mg/kg	systemic (s.c.)
			NLX 1 mg/kg + JZL184 8 mg/kg	systemic (s.c.) + systemic (i.p.)
Intra-NAcC naloxone after systemic administration of JZL184 (Figures 2E,F)	Intra-NAcC infusion of the OR antagonist naloxone blocked the play-enhancing effects of systemic treatment with JZL184 in rats	Rats	Control	Intra NAcC
			JZL184 1 mg/kg	systemic (i.p.)
			NLX 0.5 μg	Intra NAcC
			NLX 0.5 μg + JZL184 1 mg/kg	Intra NAcC + systemic (i.p.)

Because of its reciprocal nature, we studied social behavior as a dyadic interaction, i.e., we considered a pair of animals as an experimental unit. Therefore, we used pairs of animals of the same sex, age, and weight and both animals in a pair received the same treatment.

### Physiology

#### Slice preparation

Animals were anesthetized with halothane (rats) or isoflurane (mice) and decapitated according to institutional regulations. The brain was sliced (300 μm) in the coronal plane with a vibratome (Integraslice, Campden Instruments, Loughborough, UK) in a sucrose-based solution at 4°C (in mM: 87 NaCl, 75 sucrose, 25 glucose, 2.5 KCl, 4 MgCl_2_, 0.5 CaCl_2_, 23 NaHCO_3_, and 1.25 NaH_2_PO_4_). Immediately after cutting, slices were stored for 1 h at 32°C in a low calcium artificial cerebrospinal fluid (low Ca^2+^ ACSF) that contained in mM: 130 NaCl, 11 Glucose, 2.5 KCl, 2.4 MgCl_2_, 1.2 CaCl_2_, 23 NaHCO_3_, 1.2 NaH_2_PO_4_, and was equilibrated with 95% O_2_/5% CO_2_ and then at room temperature until the time of recording.

#### Electrophysiology

Whole cell patch-clamp of visualized medium spiny neurons (MSN) and field potential recordings were made in coronal slices containing the ventral striatum as previously described (Robbe et al., 2002). Recordings were made in the medial ventral NAcC close to the anterior commissure (Robbe et al., 2002). For extracellular field experiments (fEPSP), the recording pipette was filled with ACSF. Both the fEPSP amplitude and area were measured (graphs depict amplitudes). For recording, slices were placed in the recording chamber and superfused (1.5–2 ml/min) with ACSF (same as low Ca^2+^ ACSF with the following exception: 2.4 mM CaCl_2_ and 1.2 mM MgCl_2_). All experiments were done at 32°C. Stimulation was performed with a glass electrode filled with ACSF and placed ~200 μm in the dorsal-medial direction of the recorded cell. The stimulus (100 μs duration) intensity was adjusted around 60% of maximal intensity after performing an input-output curve (baseline EPSC amplitudes ranged between 50 and 150 pA). Stimulation frequency was set at 0.1 Hz. The extracellular fEPSP was confirmed to be glutamatergic by application at the end of the experiments of the non-NMDA ionotropic glutamate receptor antagonist, DNQX (20 μM), completely blocking the synaptic component (Robbe et al., 2001, 2002, 2003). The superfusion medium contained picrotoxin (100 μM) to block gamma-aminobutyric acid type A (GABA-A) receptors. All drugs were added at the final concentration to the superfusion medium. For whole cell patch-clamp experiments, neurons were visualized using an upright microscope with infrared illumination. The intracellular solution was based on K^+^ gluconate (in mM: 145 K^+^ gluconate, 3 NaCl, 1 MgCl_2_, 1 EGTA, 0.3 CaCl_2_, 2 ${\text{Na}}_{2}^{+}$ATP, and 0.3 Na^+^ GTP, 0.2 cAMP, buffered with 10 HEPES). Electrode resistance was 4–6 MOhms. Whole cell patch-clamp recordings were performed with an Axopatch-200B amplifier. Data were low pass filtered at 2 kHz, digitized (10 kHz, DigiData 1440A, Axon Instrument), collected using Clampex 10.2 and analyzed using Clampfit 10.2 (all from Molecular Device, Sunnyvale, USA). A −2 mV hyperpolarizing pulse was applied before each evoked EPSC in order to evaluate the access resistance and those experiments in which this parameter changed >25% were rejected. Access resistance compensation was not used and acceptable access resistance was <30 MOhms. The potential reference of the amplifier was adjusted to zero prior to breaking into the cell. Miniature EPSC were recorded from MSN clamped at −70 mV. Lidocaine (500 μM) was added to block voltage gated Na^2+^ channels and absence of response to stimuli confirmed for a minimum of 10 min before recording used to calculate mEPSC amplitude and frequency (Robbe et al., 2001).

#### Drugs

The GABA-A receptor antagonist picrotoxin, the MOR agonist DAMGO ([D-Ala2, N-MePhe4, Gly-ol]-enkephalin) and the MOR antagonist CTAP were from Sigma (France). The non-NMDA ionotropic glutamate receptor antagonist DNQX (6,7-dinitroquinoxaline-2,3-dione), the CB1R agonist CP55940 [2-[(1R,2R,5R)-5-hydroxy-2-(3-hydroxypropyl) cyclohexyl]-5-(2-methyloctan-2-yl)phenol], the CB1R antagonist/inverse antagonist AM251 [1-(2,4-dichlorophenyl)-5-(4-iodophenyl)-4-methyl-N-(1-piperidyl)pyrazole-3-carboxamide], the OR antagonist naloxone and the Na^+^ channel blocker lidocaine were from Tocris (Bristol, UK). Other chemicals were of the highest commercial grade available.

### Statistical analysis

All values are given as mean ±S.E.M. For electrophysiology experiments, N corresponds to the number of animals tested for each condition; for the behavioral experiments, N corresponds to the pair of animals per treatment group. Electrophysiological data were analyzed using Clampfit 10 (Molecular Devices, Sunyvale, USA). The magnitude of plasticity was calculated by 30–40 min after drug injection as percentage of baseline responses. Behavioral data were analyzed using either one-way or two-way ANOVA, followed by Student-Newman-Keuls *post-hoc* test. Statistical analyses were performed using GraphPad Prism 5 (GraphPad Software, Inc., La Jolla, CA, USA) and IBM SPSS Statistics 20 (IBM, New York, NY, USA). Differences were considered significant for *p* < 0.05.

## Results

### 2-AG acting on CB1R located in the NAcC stimulates social play in adolescent rats

Systemic administration of the 2-AG hydrolysis inhibitor JZL184 markedly increased social play in adolescent rats [one-way ANOVA: pinning, *F*_(2, 21)_ = 13.981, *p* < 0.001, Figure 1A; pouncing, *F*_(2, 21)_ = 10.190, *p* < 0.001; Figure 1B], without affecting social exploration (Table 2). *Post-hoc* analysis revealed that low doses of JZL184 (1 mg/kg) increased pinning (Figure 1A) and pouncing (Figure 1B) frequency. These play-enhancing effects were blocked following systemic pretreatment with the CB1R antagonist SR141716 (0.1 mg/kg) 30 min before systemic JZL184 [1 mg/kg; two-way ANOVA: pinning, *F*_(JZLxSR)(1, 23)_ = 6.362, *p* < 0.05; Figure 1C; pouncing, *F*_(JZLxSR)(1, 23)_ = 7.749, *p* < 0.05; Figure 1D; for complete statistical analysis see Table 3]. *Post-hoc* analysis revealed that JZL184 (1 mg/kg) increased social play when co-administered with vehicle but not when the animals were pretreated with SR141716. The most parsimonious interpretation of these data is that 2-AG acts on CB1R to stimulate social play.

**Table 2:** **Statistical report of social exploration duration in social play behavior in adolescent rats**.

**Treatment**	**No. of animals for group**	**Mean**	**SEM**	**Test**	**ANOVA *P*-value**		
JZL184 systemic administration (Figures 1A,B)	Control (*n* = 8)	248.581	24.130	One-way ANOVA	*F* = 0.485 *P* = 0.621		
	JZL184 1 mg (*n* = 9)	239.708	18.490				
	JZL184 5 mg (*n* = 7)	269.467	21.921				
JZL184 + SR141716 systemic administration (Figures 1C,D)	Control (*n* = 7)	183.630	11.242	Two-way ANOVA	**Treat_SR_**	**Treat_JZL184_**	**Treat_SRxJZL184_**
	JZL184 1 mg (*n* = 7)	228.161	22.462		*F* = 2.060	*F* = 4.553	*F* = 0.633
	SR141716A 0.1 mg (*n* = 8)	173.905	8.625		*P* = 0.165	*P* < 0.05	*P* = 0.434
	SR 0.1 mg + JZL184 1 mg (*n* = 5)	194.248	14.400				
Intra-NAcC SR141716 after systemic administration of JZL184 (Figures 1E,F)	Control (*n* = 7)	202.137	10.253	Two-way ANOVA	**Treat_SRcore_**	**Treat_JZL184*ip*_**	**Treat_SRcoreXJZL184ip_**
	JZL184 1 mg (*n* = 7)	195.491	20.738		*F* = 0.798	*F* = 4.381	*F* = 4.871
	SR141716A 3 μg (*n* = 6)	211.063	24.792		*P* = 0.381	*P* < 0.05	*P* < 0.05
	SR 3 μg + JZL184 1 mg (*n* = 7)	209.801	19.074				
Intra-NAcC SR141716 after systemic administration of morphine (Figures 2A,B)	Control (*n* = 7)	232.620	19.402	Two-way ANOVA	**Treat_SRcore_**	**Treat_MOR*sc*_**	**Treat_SRcorexMORsc_**
	MOR 1 mg (*n* = 11)	216.075	9.496		*F* = 1.454	*F* = 0.299	*F* = 3.498
	SR141716A 3 μg (*n* = 7)	194.173	12.541		*P* = 0.236	*P* = 0.588	*P* = 0.070
	SR 3 μg + MOR 1 mg (*n* = 12)	224.378	9.727				
JZL184 + naloxone systemic administration (Figures 2C,D)	Control (*n* = 8)	220.165	25.863	Two-way ANOVA	**Treat_NLX_**	**Treat_JZL184_**	**Treat_NLXxJZL184_**
	JZL184 1 mg (*n* = 9)	239.708	18.490		*F* = 2.282	*F* = 0.005	*F* = 0.909
	NLX 1 mg (*n* = 5)	209.500	12.638		*P* = 0.143	*P* = 0.946	*P* = 0.349
	NLX 1 mg + JZL184 1 mg (*n* = 8)	192.552	6.848				
Intra-NAcC naloxone after systemic administration of JZL184 (Figures 2E,F)	Control (*n* = 8)	249.316	11.744	Two-way ANOVA	**Treat_NLXcore_**	**Treat_JZL184ip_**	**Treat_NLXcorexJZL184ip_**
	JZL184 1 mg (*n* = 5)	246.668	8.390		*F* = 0.220	*F* = 0.080	*F* = 0.003
	NLX 0.5 μg (*n* = 7)	255.579	9.650		*P* = 0.644	*P* = 0.780	*P* = 0.953
	NLX 0.5 μg + JZL184 1 mg (*n* = 7)	251.529	13.940				
Intra-NAcC CTAP after systemic administration of JZL184 (Figures 3A,B)	Control (*n* = 9)	184.429	7.776	Two-way ANOVA	**Treat_CTAPcore_**	**Treat_JZL184ip_**	**Treat_CTAPcoreXJZL184ip_**
	JZL184 1 mg (*n* = 7)	219.241	11.026		*F* = 1.772	*F* = 0.9339	*F* = 2.894
	CTAP 3 μg (*n* = 10)	193.263	11.840		*P* = 0.193	*P* = 0,341	*P* = 0.099
	SR 3 μg + JZL184 1 mg (*n* = 9)	170.723	15.160				

**Table 3:** **Statistical report of social behavior parameters in adolescent rats and mice**.

**Treatment**	**Social play parameters**	**No. of animals for group**	**Mean**	**SEM**	**Test**	**ANOVA *F*- and *P*-values**		
JZL184 + SR141716 systemic administration (Figures 1C,D)	Pinning (frequency)	Control (*n* = 7)	35.9	1.8	Two-way ANOVA	**Treat_SR_**	**Treat_JZL184_**	**Treat_SRxJZL184_**
		JZL184 1 mg/kg (*n* = 7)	58.9	6.4		*F* = 8.194	*F* = 7.604	*F* = 6.362
		SR141716 0.1 mg/kg (*n* = 8)	34.4	3.6		*P* < 0.01	*P* < 0.05	*P* < 0.05
		SR 0.1 mg/kg + JZL 1 mg/kg (*n* = 5)	35.4	3.8				
	Pouncing (frequency)	Control (*n* = 7)	60.3	4.4	Two-way ANOVA	**Treat_SR_**	**Treat_JZL184_**	**Treat_SR x JZL184_**
		JZL184 1 mg/kg (*n* = 7)	100.3	8.3		*F* = 7.563	*F* = 12.176	*F* = 7.749
		SR141716 0.1 mg/kg (*n* = 8)	60.5	3.9		*P* < 0.05	*P* < 0.01	*P* < 0.05
		SR 0.1 mg/kg + JZL 1 mg/kg (*n* = 5)	65.0	8.8				
Intra-NAcC SR141716 after systemic administration of JZL184 (Figures 1E,F)	Pinning (frequency)	Control (*n* = 7)	30.7	4.4	Two-way ANOVA	**Treat_SRNAcC_**	**Treat_JZL184ip_**	**Treat_SRNAcC x JZL184ip_**
		JZL184 1 mg/kg (*n* = 7)	52.9	3.3		*F* = 3.748	*F* = 7.399	*F* = 5.496
		SR141716 3 μg (*n* = 6)	32.5	5.7		*P* = 0.06	*P* < 0.05	*P* < 0.05
		SR 3 μg + JZL184 1 mg/kg (*n* = 7)	34.1	4.1				
	Pouncing (frequency)	Control (*n* = 7)	67.6	9.2	Two-way ANOVA	**Treat_SRNAcC_**	**Treat_JZL184ip_**	**Treat_SRNAcC x JZLip_**
		JZL184 1 mg/kg (*n* = 7)	104.4	6.6		*F* = 0.798	*F* = 4.381	*F* = 4.871
		SR141716 3 μg (*n* = 6)	78.8	5.4		*P* = 0.381	*P* < 0.05	*P* < 0.05
		SR 3 μg + JZL184 1 mg/kg (*n* = 7)	77.9	11.0				
Intra-NAcC SR141716 after systemic administration of morphine (Figures 2A,B)	Pinning (frequency)	Control (*n* = 7)	40.3	8.3	Two-way ANOVA	**Treat_SRNAcC_**	**Treat_MORsc_**	**Treat_SRNAcC x MORsc_**
		MOR 1 mg/kg (*n* = 11)	81.5	11.1		*F* = 6.029	*F* = 3.202	*F* = 8.694
		SR141716A 3 μg (*n* = 7)	44.6	5.3		*P* < 0.05	*P* = 0.08	*P* < 0.01
		SR 3 μg + MOR 1 mg/kg (*n* = 12)	34.5	5.6				
	Pouncing (frequency)	Control (*n* = 7)	73.4	6.2	Two-way ANOVA	**Treat_SRNAcC_**	**Treat_MORsc_**	**Treat_SRNAcC x MORsc_**
		MOR 1 mg/kg (*n* = 11)	131.2	13.1		*F* = 9.611	*F* = 5.795	*F* = 8.882
		SR141716 3 μg (*n* = 7)	72.1	10.6		*P* < 0.01	*P* < 0.05	*P* < 0.01
		SR 3 μg + MOR 1 mg/kg (*n* = 12)	66.0	7.7				
JZL184 + naloxone systemic administration (Figures 2C,D)	Pinning (frequency)	Control (*n* = 8)	18.6	3.5	Two-way ANOVA	**Treat_NLX_**	**Treat_JZL184_**	**Treat_NLX x JZL184_**
		JZL184 1 mg /kg(*n* = 9)	40.8	2.8		*F* = 3.277	*F* = 17.918	*F* = 5.165
		NLX 1 mg/kg (*n* = 5)	20.2	4.6		*P* = 0.082	*P* < 0.001	*P* < 0.05
		NLX 1 mg/kg + JZL 1 mg/kg (*n* = 8)	26.9	3.0				
	Pouncing (frequency)	Control (*n* = 8)	53.1	4.3	Two-way ANOVA	**Treat_NLX_**	**Treat_JZL184_**	**Treat_NLX x JZL184_**
		JZL184 1 mg/kg (*n* = 9)	79.4	6.0		*F* = 2.147	*F* = 5.949	*F* = 7.223
		NLX 1 mg/kg (*n* = 5)	59.4	6.8		*P* = 0.155	*P* < 0.05	*P* < 0.05
		NLX 1 mg/kg + JZL 1 mg/kg (*n* = 8)	58.1	2.4				
Intra-NAcC naloxone after systemic administration of JZL184 (Figures 2E,F)	Pinning (frequency)	Control (*n* = 8)	43.9	3.5	Two-way ANOVA	**Treat_NLXNAcC_**	**Treat_JZL184ip_**	**Treat_NLXNAcC x JZLip_**
		JZL184 1 mg (*n* = 5)	72.0	9.6		*F* = 7.871	*F* = 4.452	*F* = 5.239
		NLX 0.5 μg (*n* = 7)	40.6	6.5		*P* < 0.05	*P* < 0.05	*P* < 0.05
		NLX 0.5 μg + JZL184 1 mg (*n* = 7)	39.4	6.6				
	Pouncing (frequency)	Control (*n* = 8)	85.3	5.7	Two-way ANOVA	**Treat_NLXNAcC_**	**Treat_JZL184ip_**	**Treat_NLXNAcC x JZLip_**
		JZL184 1 mg/kg (*n* = 5)	121.0	8.5		*F* = 15.920	*F* = 2.881	*F* = 5.856
		NLX 0.5 μg (*n* = 7)	77.1	7.0		*P* < 0.001	*P* = 0.10	*P* < 0.05
		NLX 0.5 μg + JZL 1 mg/kg (*n* = 7)	71.6	9.4				
Intra-NAcC CTAP after systemic administration of JZL184 (Figures 3A,B)	Pinning (frequency)	Control (*n* = 9)	38.9	5.9	Two-way ANOVA	**Treat_CTAPNAcC_**	**Treat_JZL184ip_**	**Treat_CTAPNAcC x JZLip_**
		JZL184 1 mg/kg (*n* = 7)	69.0	8.3		*F* = 9.812	*F* = 2.627	*F* = 2.845
		CTAP 3 μg (*n* = 10)	39.3	4.1		*P* < 0.01	*P* = 0.12	*P* = 0.10
		SR 3 μg + JZL184 1 mg/kg (*n* = 9)	48.3	7.0				
	Pouncing (frequency)	Control (*n* = 9)	71.2	8.7	Two-way ANOVA	**Treat_*CTAPNAcC*_**	**Treat_JZL184ip_**	**Treat_CTAPNAcC x JZLip_**
		JZL184 1 mg/kg (*n* = 7)	114.9	10.4		*F* = 5.851	*F* = 1.103	*F* = 7.030
		CTAP 3 μg (*n* = 10)	85.0	9.1		*P* < 0.05	*P* = 0.30	*P* < 0.05
		SR 3 μg + JZL184 1 mg/kg (*n* = 9)	83.0	5.6				
JZL184 + SR141716 systemic administration (Figures 4C,D)	Duration (s)	Control (*n* = 8)	101.8	7.6	Two-way ANOVA	**Treat_SR_**	**Treat_JZL184_**	**Treat_SR x JZL184_**
		JZL184 8 mg/kg (*n* = 8)	133.2	9.5		*F* = 2.516	*F* = 0.587	*F* = 6.679
		SR141716 3 mg/kg (*n* = 6)	111.1	5.3		*P* = 0.125	*P* = .451	*P* < 0.05
		SR 3 mg/kg + JZL 8 mg/kg (*n* = 7)	94.1	12.5				
	Frequency	Control (*n* = 8)	40.4	1.8	Two-way ANOVA	**Treat_SR_**	**Treat_JZL184_**	**Treat_SR x JZL184_**
		JZL184 8 mg/kg (*n* = 8)	54.0	3.2		*F* = 1.924	*F* = 1.747	*F* = 6.231
		SR141716 3 mg/kg (*n* = 6)	44.3	2.8		*P* = 0.177	*P* = 0.198	*P* < 0.05
		SR 3 mg/kg + JZL 8 mg/kg (*n* = 7)	40.1	5.5				
JZL184 + naloxone systemic administration (Figures 4E,F)	Duration (s)	Control (*n* = 6)	109.6	9.5	Two-way ANOVA	**Treat_NLX_**	**Treat_JZL184_**	**Treat_NLX x JZL184_**
		JZL184 8 mg/kg (*n* = 6)	152.8	12.5		*F* = 0.254	*F* = 1.968	*F* = 6.717
		NLX 1 mg/kg (*n* = 8)	115.6	9.7		*P* = 0.619	*P* = 0.173	*P* < 0.05
		NLX 1 mg/kg + JZL 8 mg/kg (*n* = 9)	108.0	9.8				
	Frequency	Control (*n* = 6)	55.8	3.0	Two-way ANOVA	**Treat_NLX_**	**Treat_JZL184_**	**Treat_NLX x JZL184_**
		JZL184 8 mg/kg (*n* = 6)	73.3	4.7		*F* = 0.627	*F* = 1.091	*F* = 3.883
		NLX 1 mg/kg (*n* = 8)	48.5	3.1		*P* = 0.435	*P* = 0.305	*P* < 0.05
		NLX 1 mg/kg + JZL 8 mg/kg (*n* = 9)	48.7	3.7				

Next, we tested whether the effect of JZL184 on social play was mediated through the NAcC. We found that intra-NAcC infusion of SR141716 antagonized the play-enhancing effects of systemic JZL184 [1 mg/kg; two-way ANOVA: pinning, *F*_(JZLsyst×SR NAcC)(1, 23)_ = 5.496, *p* < 0.05; Figure 1E; pouncing, *F*_(JZLsyst x SR NAcC)(1, 23)_ = 4.871, *p* < 0.05; Figure 1F; for complete statistical analysis see Table 3]. *Post-hoc* analysis showed that 2-AG elevation stimulates social play in adolescent rats via CB1R located in the NAcC. Thus, elevation of 2-AG stimulates social play by activating CB1R located in the NAcC.

### MOR stimulation in the NAcC is necessary for the play-enhancing effect of JZL184

Our previous work has shown that systemic CB1R antagonism reduced the social play-enhancing effects of the MOR agonist morphine (Trezza and Vanderschuren, 2008a). In light of the present results, we tested whether intra-NAcC CB1R are involved in the increase in social play induced by systemic administration of morphine. Strikingly, we found that the morphine-induced increase in social play was prevented by NAcC CB1Rs [two-way ANOVA: pinning, *F*_(MORsyst×SR NAcC)(1, 33)_ = 8.694, *p* < 0.01; Figure 2A; pouncing, *F*_(MORsyst×SR NAcC)(1, 33)_ = 8.882, *p* < 0.01; Figure 2B; for complete statistical analysis see Table 3]. *Post-hoc* analysis showed that the play-enhancing effects of systemic administration of morphine (1 mg/kg) were prevented by the intra-NAcC infusion of a dose of SR141716 that did not affect social play by itself.

**Figure 3:**
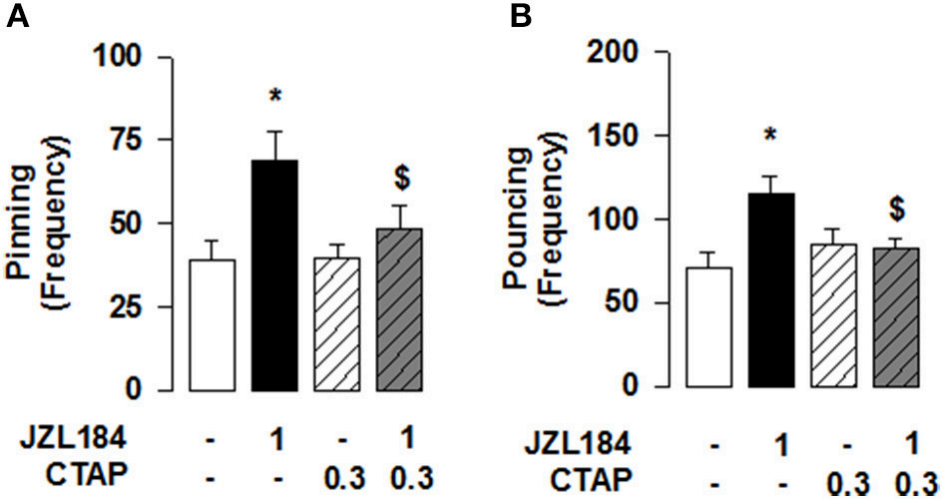
**2-AG elevation stimulates social play via MOR in the NAcC in adolescent rats**. Intra-NAcC infusion of the selective MOR antagonist CTAP (0.3 μg/0.3 μl) prevented the increase in pinning **(A)** and pouncing **(B)** induced by systemic treatment with JZL184 (1 mg/kg, i.p.). Data represent mean ± S.E.M. frequency of pinning and pouncing. ^*^p < 0.05 vs. vehicle; ^*$*^*p* < 0.05 vs. vehicle/JZL184 (Student-Newman-Keuls *post-hoc* test). *N* = 7–9 per treatment group.

Conversely, we tested if 2-AG-induced increase in social play required MOR in the NAcC. First, we observed that systemic pretreatment with the MOR antagonist naloxone (1 mg/kg) 30 min before systemic administration of JZL184 (1 mg/kg) prevented the play-enhancing effects of systemic administration of JZL184 [two-way ANOVA: pinning, *F*_(JZLxNLX)(1, 26)_ = 5.165, *p* < 0.05; Figure 2C; pouncing, *F*_(JZLxNLX)(1, 26)_ = 7.223, *p* < 0.05; Figure 2D; for complete statistical analysis see Table 3]. *Post-hoc* analysis revealed that JZL184 (1 mg/Kg) increased social play when co-administered with vehicle but not when the animals were pretreated with naloxone. In support of our working hypothesis, we found that intra-NAcC infusion of naloxone antagonized increase in social play induced by systemic treatment with JZL184 (1 mg/kg) [two-way ANOVA: pinning, *F*_(JZLsyst×NLX NAcC)(1, 23)_ = 5.239, *p* < 0.05; Figure 2E; pouncing, *F*_(JZLsyst×NLX NAcC)(1, 23)_ = 5.856, *p* < 0.05; Figure 2F; for complete statistical analysis see Table 3]. *Post-hoc* analysis showed that JZL184-induced increase in social play behavior was absent in rats that received intra-NAcC naloxone. Naloxone is only moderately selective for MORs (Goldstein and Naidu, 1989; Mansour et al., 1995). Therefore, to test whether the play-enhancing effects of JZL184 were specifically mediated by MORs, animals received systemic administration of JZL184 followed by intra-NAcC infusion of the selective MOR antagonist CTAP. Intra-NAcC infusion of CTAP inhibited the effects of systemic JZL184 treatment on social play [two-way ANOVA: pinning, *F*_(JZLsyst×CTAP NAcC)(1, 31)_ = 2.845, *p* = 0.10; Figure 3A; pouncing, *F*_(JZLsyst×CTAP NAcC)(1, 31)_ = 7.030, *p* < 0.05; Figure 3B; for complete statistical analysis see Table 3]. *Post-hoc* analysis showed that the play-enhancing effects of systemic administration of JZL184 were prevented by intra-NAcC infusion with a non-effective dose of CTAP.

### 2-AG signaling is involved in social behavior via CB1Rs and MORs in adolescent mice

**Figure 4:**
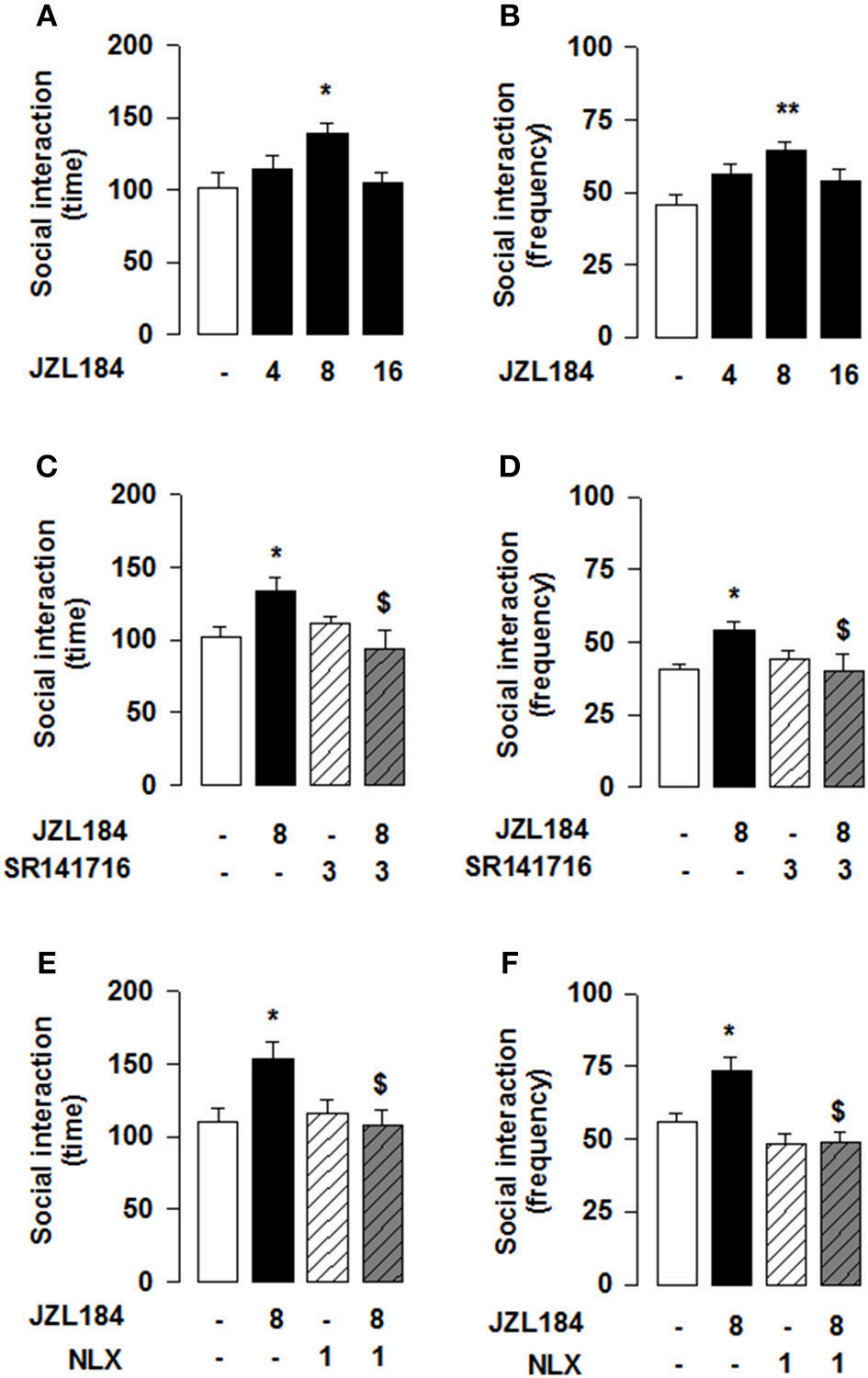
**2-AG elevation in adolescent mice stimulates social behavior through CB1R and OR**. JZL184 (8 mg/kg, i.p.) increased the total time **(A)** and frequency **(B)** of social interaction. Pre-treatment with the CB1R antagonist SR141716 (3 mg/kg, i.p.) antagonized these effects **(C,D)**. In addition, pre-treatment with the OR antagonist naloxone (1 mg/kg, s.c.) prevented the effects of systemic JZL184 on social behavior (8 mg/kg, i.p.) **(E,F)**. Data represent mean ± S.E.M. time **(A,C,E)** and frequency **(B,D,F)** of social interaction. ^*^p < 0.05, ^**^p < 0.01 vs. vehicle; ^*$*^*p* < 0.05 vs. vehicle/JZL184 (Student-Newman-Keuls *post-hoc* test). *N* = 7–9 per treatment group.

**Figure 5:**
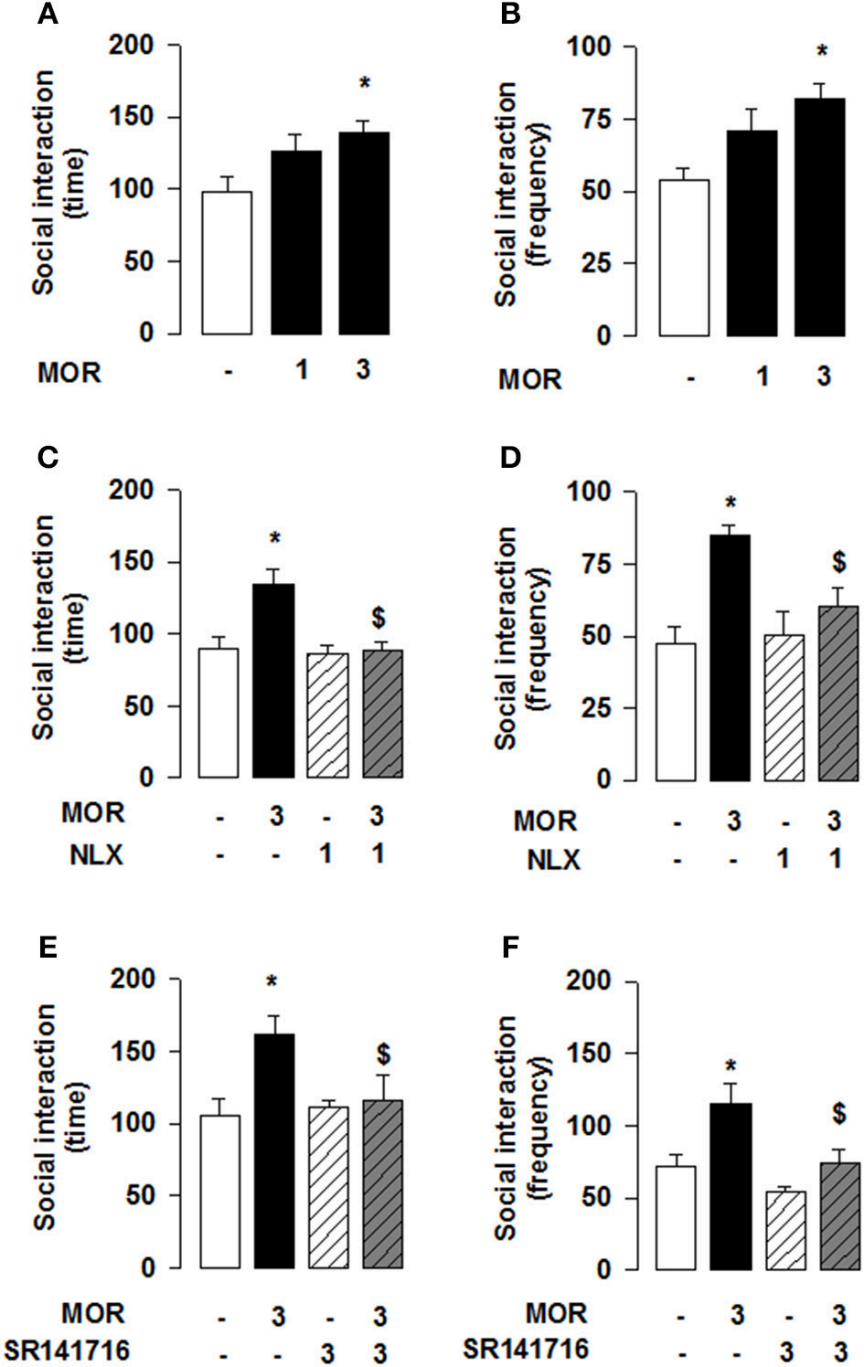
**Morphine stimulates social play in adolescent mice via OR and CB1R activation**. The OR agonist morphine (3 mg/kg, s.c.) increased the total time **(A)** and frequency **(B)** of social interaction. Pre-treatment with the OR antagonist naloxone (1 mg/kg, s.c.) prevented the social interaction-enhancing effects of systemic morphine **(C,D)**. Likewise, pre-treatment with the CB1R antagonist SR141716 (3 mg/kg, i.p.) antagonized morphine's effects on social interaction **(E,F)**. Data represent mean ± S.E.M. time **(A,C,E)** and frequency **(B,D,F)** of social interaction. ^*^p < 0.05 vs. vehicle; ^*$*^*p* < 0.05 vs. vehicle/morphine (Student-Newman-Keuls *post-hoc* test). *N* = 7–10 per treatment group.

Reciprocal opioid-eCB antagonism in the modulation of social behavior also exists in adolescent mice. Systemic administration of JZL184 to young mice increased social interaction [one-way ANOVA: total time [*F*_(3, 27)_ = 3.786, *p* < 0.05; Figure 4A] and frequency [*F*_(3, 27)_ = 4.693, *p* < 0.01; Figure 4B] of social interaction]. *Post-hoc* analysis revealed that, at the dose of 8 mg/kg, JZL184 increased the total time (Figure 4A) and frequency (Figure 4B) of social interaction in adolescent mice. Pretreatment with the CB1R antagonist SR141716 (3 mg/kg) 30 min before systemic administration of JZL184 (8 mg/kg) prevented the increase in social behavior induced by treatment with JZL184 [two-way ANOVA: total time [*F*_(JZLxSR)(1, 25)_ = 6.679, *p* < 0.05; Figure 4C] and frequency [*F*_(JZLxSR)(1, 25)_ = 6.231, *p* < 0.05; Figure 4D] of social interaction; for complete statistical analysis see Table 3]. *Post-hoc* analysis revealed that JZL184 increased social behavior when co-administered with vehicle but not in animals pretreated with SR141716.Pretreatment with naloxone (1 mg/kg) also antagonized the effects of systemic JZL184 (8 mg/kg) [two-way ANOVA: total time [*F*_(JZLxSR)(1, 25)_ = 6.717 *p* < 0.05; Figure 4E] and frequency [*F*_(JZLxSR)(1, 25)_ = 3.883, *p* < 0.05; Figure 4F] of social interaction; for complete statistical analysis see Table 3]: thus, JZL184 increased social interaction in mice pre-treated with vehicle but not in animals that received naloxone. This interaction was bidirectional since the social interaction-enhancing effects of morphine were blocked by systemic administration of naloxone or SR141716 (Figure 5; for statistical analysis see Table 4).

**Table 4:** **Statistical report of social behavior parameters in adolescent mice following systemic morphine**.

**Treatment**	**Social interaction parameter**	**No. of animals for group**	**Mean**	**SEM**	**Test**	**ANOVA *P*-value**		
Morphine systemic administration (Figures 5A,B)	Duration (s)	Control (*n* = 5)	97.808	10.394	One-way ANOVA	*F* = 3.798 *P* < 0.05		
		MOR 1 mg (*n* = 7)	126.766	11.079				
		MOR 3 mg (*n* = 6)	138.747	8.328				
	Frequency	Control (*n* = 5)	54.000	3.795	One-way ANOVA	*F* = 4.739 *P* < 0.05		
		MOR 1 mg (*n* = 7)	71.000	7.270				
		MOR 3 mg (*n* = 6)	82.000	5.465				
Morphine + naloxone systemic administration (Figures 5C,D)	Duration (s)	Control (*n* = 7)	90.177	7.326	Two-way ANOVA	**Treat_NLX_**	**Treat_MOR_**	**Treat_NLXxMOR_**
		MOR 3 mg (*n* = 6)	134.640	10.501		*F* = 9.973	*F* = 8.904	*F* = 7.173
		NLX 1 mg (*n* = 6)	86.410	6.157		*P* < 0.01	*P* < 0.01	*P* < 0.05
		MOR 3 mg + NLX 1mg (*n* = 5)	88.810	5.456				
	Frequency	Control (*n* = 7)	47.571	5.584	Two-way ANOVA	**Treat_NLX_**	**Treat_MOR_**	**Treat_NLXxMOR_**
		MOR 3mg (*n* = 6)	84.833	3.851		*F* = 3.067	*F* = 14.634	*F* = 4.830
		NLX 1mg (*n* = 6)	50.333	7.999		*P* = 0.09	*P* < 0.01	*P* < 0.05
		MOR 3mg + NLX 1mg (*n* = 5)	60.400	6.539				
Morphine + SR141716 systemic administration (Figures 5E,F)	Duration (s)	Control (*n* = 6)	105.093	11.579	Two-way ANOVA	**Treat_SR_**	**Treat_MOR_**	**Treat_SRxMOR_**
		MOR 3 mg (*n* = 6)	161.427	13.114		*F* = 2.243	*F* = 5.555	*F* = 3.938
		SR141716 3 mg (*n* = 6)	111.117	5.315		*P* = 0.149	*P* < 0.05	*P* < 0.05
		MOR 3 mg + SR 3 mg (*n* = 7)	116.309	17.066				
	Frequency	Control (*n* = 6)	71.833	7.888	Two-way ANOVA	**Treat_SR_**	**Treat_MOR_**	**Treat_SRxMOR_**
		MOR 3 mg (*n* = 6)	115.167	14.460		*F* = 8.948	*F* = 10.51	*F* = 1.378
		SR141716 3 mg (*n* = 6)	54.000	3.642		*P* < 0.01	*P* < 0.01	*P* = 0.253
		MOR 3 mg + SR 3 mg (*n* = 7)	74.286	9.817				

### Inhibitory CB1R and MOR interact in the NAcC of adolescent rats and mice

**Figure 6:**
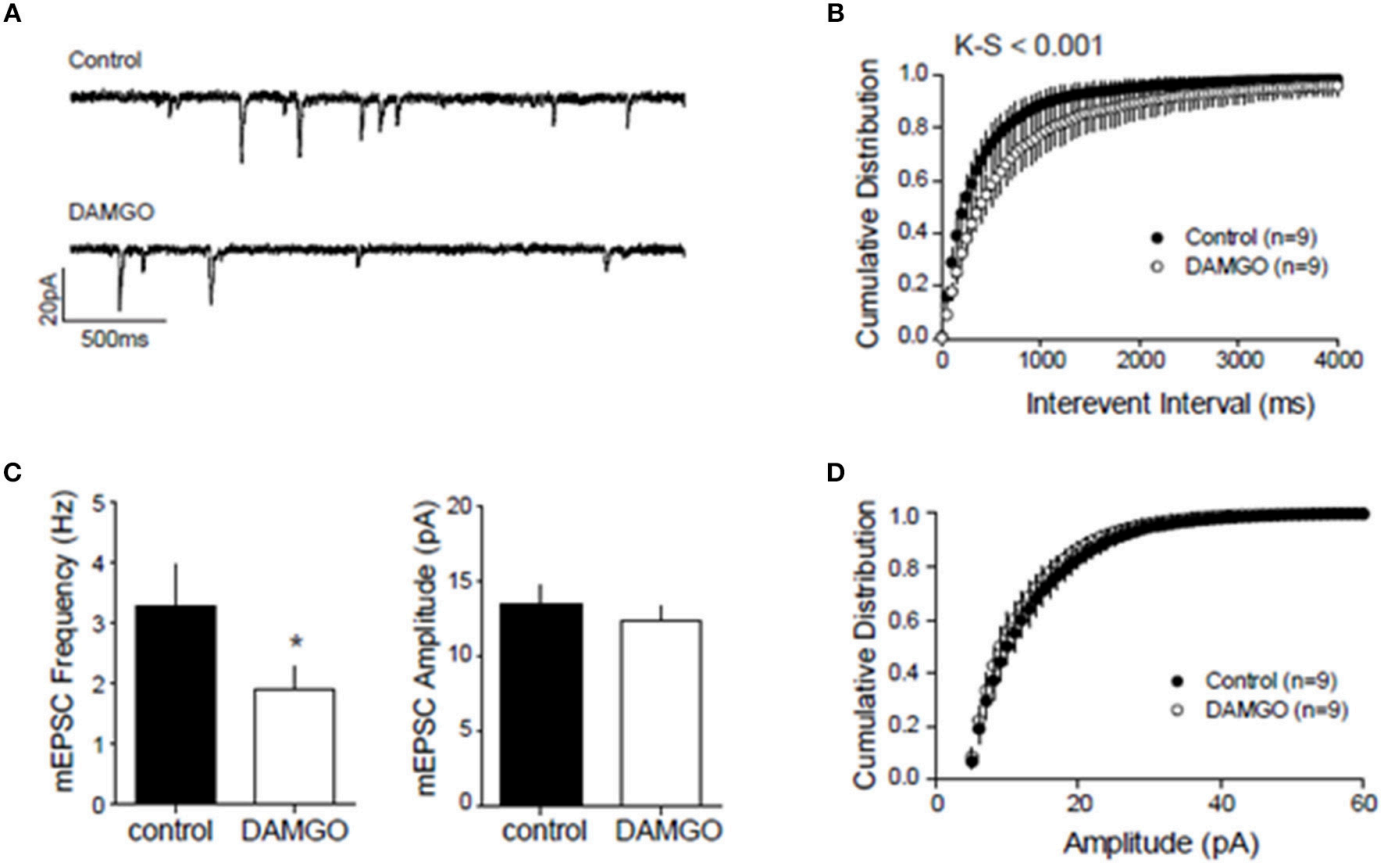
**Presynaptic inhibitory CB1 and MOR interact at excitatory NAcC synapses**. To localize the inhibitory action of DAMGO on excitatory NAcC synapses, we recorded miniature EPSCs by whole cell recordings in NAcC medium spiny neurons (MSN) clamped at –70 mV. mEPSC frequency and amplitude were calculated in the 10-min interval before and after 20 min of bath application of 10 μM DAMGO. Effect of DAMGO on miniature excitatory events in NAcC MSN: **(A)** Representative mEPSC of recordings before (control) and after DAMGO bath application; **(B)** Cumulative probability distribution of intervals between events showing a shift to the right after DAMGO and the length of inter-event interval was markedly increased [Kolmogorov-Smirnov (K-S), *p* < 0.001]; **(C)** Lower mean mEPSC frequency but no change in mean amplitude after DAMGO (*N* = 9 for both groups, ^*^p < 0.05 by paired *t*-test); **(D)** Cumulative probability distribution of mEPSC amplitude showing no change. The cumulative distribution [(K-S) test; n.s.] and mean amplitude of the mEPSC remained unchanged after DAMGO application (CTRL: mean 13.46 ± 1.2 pA; DAMGO: mean 12.28 ± 0.99 pA; paired *t*-test). Error bars represent S.E.M.

**Figure 7:**
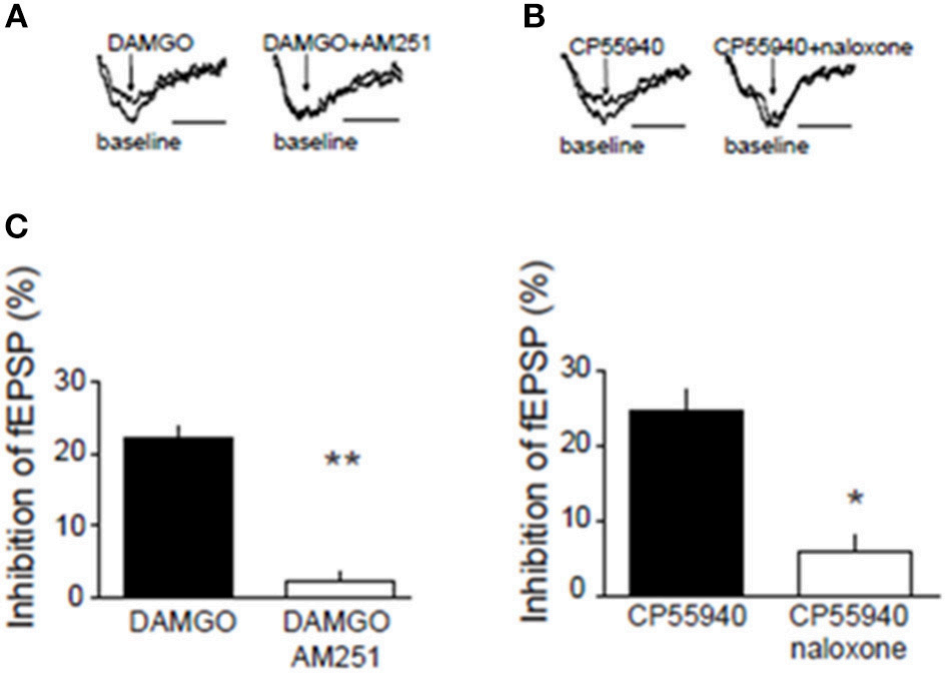
**Presynaptic inhibitory CB1R and MOR interact in the NAcC**. Representative field potential responses (fEPSP) before (baseline) and after drug application (arrow) **(A,B)**. The CB1R antagonist AM251 blocked the fEPSP inhibition induced by the selective MOR agonist DAMGO in rat NAcC slices (**C**, left panel). Conversely, the OR antagonist naloxone prevented the inhibition of fEPSP induced by the CB1R agonist CP55940 in rat NAcC slices (**C**, right panel). Data represent mean ± S.E.M. percent fEPSP inhibition after drug application. ^*^p < 0.05; ^**^p < 0.01 vs. DAMGO and CP55940. *N* = 5–8 per treatment group.

**Figure 8:**
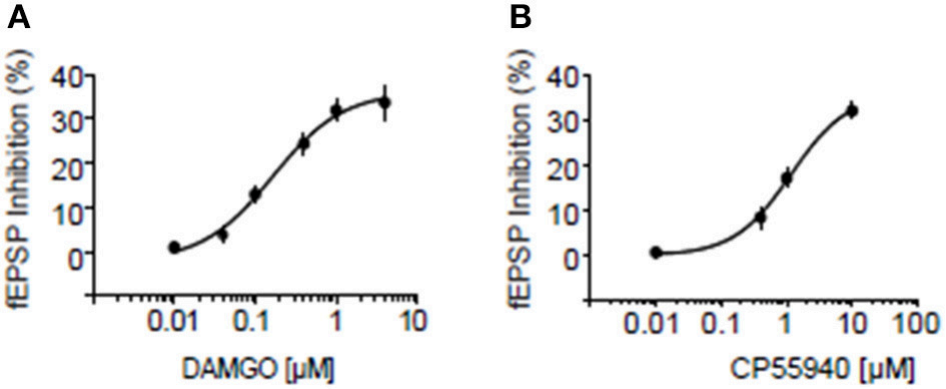
**Dose response curves for inhibition by MOR and CB1R agonists at excitatory NAcC synapses**. Dose response curve for the MOR agonist DAMGO **(A)** and for the CB1R agonist CP55940 **(B)** in rats. Error bars represent S.E.M. *N* = 3–5 per doses.

**Figure 9:**
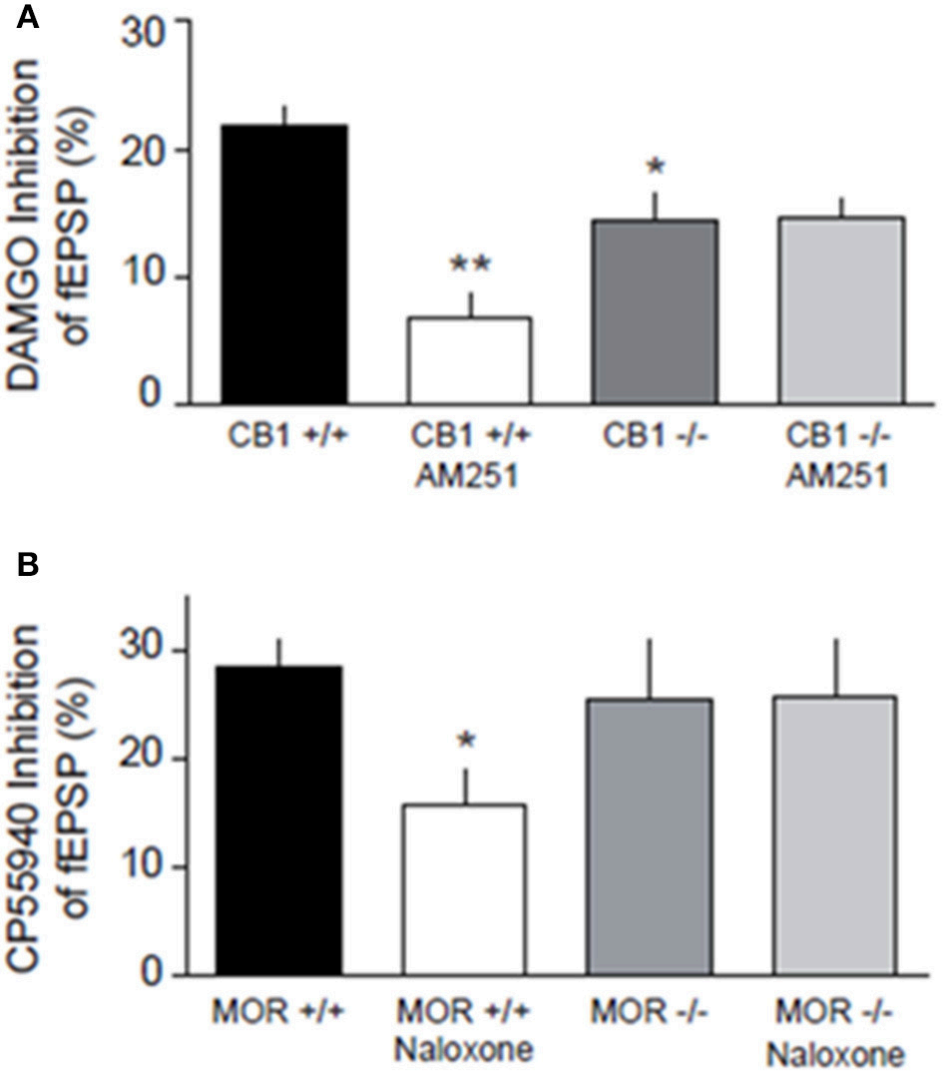
**Genetic deletion of presynaptic CB1R and MOR in the mouse alters synaptic transmission at NAcC synapses**. DAMGO-induced inhibition of fEPSP is blocked by the CB1R antagonist AM251 in CB1^+/+^, but not in CB1^−/−^ mice, and less effective in reducing excitatory transmission in CB1^−/−^ mice **(A)**. CP55940-induced inhibition of fEPSP is reduced by naloxone in MOR^+/+^, but not in MOR^−/−^ mice **(B)**. Data represent mean ± S.E.M. percent fEPSP inhibition after drug application. ^*^p < 0.05; ^**^p < 0.01 vs. DAMGO/CP55940 in CB1^+/+^ and MOR^+/+^. *N* = 5–8 per treatment group.

We next searched for synaptic underpinnings of the CB1R/MOR interaction in the NAcC. Whole cell recordings of miniature excitatory postsynaptic currents (mEPSCs) revealed that, like CB1Rs, MORs act presynaptically to inhibit excitatory transmission onto NAcC neurons (Figure 6). We next investigated the functional relationship between these two inhibitory presynaptic receptors (Figures 7, **9**). In adolescent rat NAcC slices, CB1R antagonism blocked the inhibitory effect of a maximally effective concentration of the MOR agonist DAMGO on excitatory field synaptic responses (fEPSP; *p* < 0.01; Figure 7C left panel; for DAMGO full dose-response see Figure 8A). Likewise, the maximal inhibitory effect of the CB1R agonist CP55940 on fEPSP was significantly reduced by the MOR antagonist naloxone (*p* < 0.05; Figure 7C right panel; for CP55940 full dose-response see Figure 8B). Thus, both CB1R and MOR signaling are required for the effects of both eCBs and opioids in the NacC.

We next relied on mouse genetics to test whether the function of either MORs or CB1Rs depends on the presence of the other receptor. We first verified that reciprocal opioid-cannabinoid antagonism existed in the mouse NAcC. In WT littermates of CB1R^−/−^ mice, CB1R antagonist AM251 significantly reduced DAMGO-induced inhibition of fEPSP (*p* < 0.01; Figure 9A). Likewise, the MOR antagonist naloxone reduced CP55940-induced inhibition of fEPSP in WT littermates of MOR^−/−^ mice (*p* < 0.05; Figure 9B), demonstrating that the CB1R-MOR interaction is a feature shared by mice and rats. In marked contrast with the WT phenotype, the CB1R antagonist AM251 lost its effect on the DAMGO-induced inhibition of fEPSP in CB1R^−/−^ mice (Figure 9A; n.s.), showing that opioid-induced inhibition of fEPSP occurs through CB1Rs. Interestingly, the MOR agonist DAMGO was less effective in reducing excitatory transmission in CB1R^−/−^ mice (*p* < 0.05; Figure 9A), indicating that the expression of CB1R is necessary for the full inhibitory effect of DAMGO. Likewise, the MOR antagonist naloxone did not antagonize eCB-induced inhibition of fEPSP in MOR^−/−^ mice (Figure 9B; n.s.) showing that CB1R-induced inhibition of fEPSP occurs through MORs in the NAcC.

## Discussion

The present study supports the idea that CB1R and MOR interact in the NAcC to underlie the actions of endogenous cannabinoid lipids and opioid peptides on social behavior in adolescent rats and mice. Social play is one of the earliest forms of non-mother directed social behavior observed in mammals. It contains behavioral patterns related to social, sexual and aggressive behavior (Vanderschuren et al., 1997; Trezza et al., 2010). During post-weaning development, social play is mostly directed at conspecifics and the ability to engage in social play is one of the principal indicators of healthy development. The endocannabinoid system participates in emotional homeostasis from early developmental stages onwards (Viveros et al., 2007; Solinas et al., 2008; Berridge et al., 2010; Campolongo et al., 2011). In adolescent rats, anandamide promotes social play via CB1R in the basolateral amygdala (Trezza et al., 2012) and in adult mice it mediates oxytocin-driven social reward via CB1R located in the NAc (Wei et al., 2015). The main endocannabinoid 2-AG is released in the brain of adolescent rats during social play (Manduca et al., 2015), although the exact brain region where 2-AG modulates social play was unknown. Furthermore, 2-AG levels have been shown to be higher in the NAc of socially stimulated mice compared to isolated mice (Wei et al., 2016), and 2-AG decreases aggressive behavior in a resident/intruder test in adult mice, suggesting a role in social challenge (Aliczki et al., 2014). In the present study, we found that JZL184, which produces a long-lasting elevation of brain 2-AG by inhibiting MAGL mediated 2-AG hydrolysis (Long et al., 2009; Seillier et al., 2014; Morena et al., 2015), increased the frequency of pinning and pouncing, the two principal characteristic parameters of social play in adolescent rats. Our data unequivocally demonstrate that 2-AG stimulated social play depending upon activation of CB1R in the NAcC. Thus both anandamide and 2-AG participate in social reward (Marco et al., 2011; Trezza et al., 2012; Wei et al., 2015, 2016) and social play (Trezza et al., 2012). The endogenous opioid system bidirectionally modulates social behavior in adolescent rats: accumbens MOR and κ-opioid receptors stimulate and inhibit social play, respectively (Trezza et al., 2011b). With respect to the eCB system we previously reported that the play-stimulating properties of anandamide were inhibited by opioid antagonism, and vice versa (Trezza and Vanderschuren, 2008a, 2009). Specifically, we showed that the anandamide hydrolysis inhibitor URB597 enhanced social play. This effect was blocked by pretreatment with the OR antagonist naloxone, and the well-known stimulatory effect of morphine on social play behavior was attenuated by pretreatment with the CB1R antagonist SR141716 (Trezza and Vanderschuren, 2008a, 2009). Here, we demonstrate that the 2-AG-induced increase in social play requires MOR in the NAcC: infusion of either naloxone or the selective MOR antagonist CTAP into the NAcC prevented the play-enhancing effects of JZL184. Thus, 2-AG modulates social play through stimulation of both CB1R and MOR in the NAcC. Strikingly, this interaction was bidirectional: the increase in social play induced by systemic treatment with the OR agonist morphine was blocked by intra-NAcC CB1R inhibition. These data suggest a reciprocal interaction between eCB lipid and opioid peptide systems in the NAcC to regulate social play behavior. Previous findings reported the importance of NAcC in social play (Trezza et al., 2011b; Manduca et al., 2016) and the role of opioids and eCBs in the modulation of social play in this brain structure (Trezza et al., 2011b, 2012). However, to the best of our knowledge, this is the first study showing a close relationship between CBR1 and MOR specifically in the NAcC in the modulation of social play behavior. Importantly, our present data extend the role of opioid-eCB interactions in the modulation of social interaction to social interaction in young mice. In contrast to rats, in which social play behavior is easy to distinguish from non-playful social behavior (Panksepp and Beatty, 1980; Vanderschuren et al., 1997), social play behavior is hard to recognize as such in mice (Pellis and Pasztor, 1999). Therefore, we can not say with confidence whether the nature of the social behavior in mice reported here is strictly playful. Nevertheless, the fact that social interaction in young mice is modulated by two interacting neurotransmitter systems that have been widely implicated in reward processes (Van Ree et al., 2000; Solinas et al., 2008; Le Merrer et al., 2009; Berridge and Kringelbach, 2015), strongly suggests that this social behavior in young mice has positive emotional value.

*In vivo* studies have previously shown that genetic deletion of CB1R reduces morphine self-administration and attenuates morphine-induced conditioned place preference (Chaperon et al., 1998; Ledent et al., 1999; Navarro et al., 2001; Caille and Parsons, 2003; Solinas et al., 2003), indicating that functional CB1Rs are required for the rewarding effects of opiates. Biochemical evidence of interactions between CB1Rs and MORs are abound (Shapira et al., 2000; Salio et al., 2001; Hojo et al., 2008): *in vitro* bioluminescence resonance energy transfer showed CB1R hetero-oligomerizing with MOR (Rios et al., 2006) and CB1R/MOR interaction were found in FRET and co-immunoprecipitation experiments in expression cell models (Hojo et al., 2008). Finally, neurochemical data have indicated that allosterically-interacting MOR and CB1R control neurotransmitter release in the NAcC (Schoffelmeer et al., 2006). In line with these results, we report electrophysiological evidence of opioid-cannabinoid antagonism at excitatory NAcC synapses of mice and rats where CB1R are located on axon terminals contacting NAcC medium spiny neurons (Robbe et al., 2001; Pickel et al., 2004) while MOR are found both pre- and post-synaptically (Hoffman et al., 2003; Pickel et al., 2004). Here, we found that the effects of the CB1R antagonist on opioid-induced inhibition of fEPSP were lost in CB1R^−/−^ mice while naloxone did not antagonize CB1R in MOR^−/−^ mice. Furthermore, the MOR agonist DAMGO was less efficient in CB1R^−/−^ mice, indicating that CB1R are necessary for the full inhibitory effect of DAMGO.

Collectively, the data support the idea that these two cognate presynaptic receptors interact, perhaps within a heterodimer complex, to underlie eCB and opioid effects in the NAcC and provide a plausible substrate for the heterologous interaction between MOR and CB1R in the regulation of positively valenced social behavior in rodents. Further, understanding of the neural mechanisms of rewarding social interactions may help to increase knowledge about the physiological mechanisms of adaptive social development, as well as of the mental disorders characterized by aberrant social behavior.

## Ethics statement

All experiments were performed according to INSERM ethic rules. This study and protocols were approved by the ethic committee of Marseille under the reference n°2015121715284829-V1 n°#3279.

## Author contributions

AM, MS, LV, VT, and OM designed research; AM, MS, PC, VC, and OL performed research; AM, MS, and OL analyzed data; GM and BK provided transgenic mice and help designing research; AM, LV, VT, MS, and OM wrote the paper; VT and OM supervised the entire project.

## Funding

This study was supported by the Netherlands Organization for Scientific Research (NWO) Veni grant 91611052 (to VT), Marie Curie Career Reintegration grant PCIG09-GA-2011-293589 (to VT), and the National Institute on Drug Abuse grant R01 DA022628 (to LV), The Netherlands and INSERM, INRA, ANR Presynaptic-CB1R (to GM and OM).

### Conflict of interest statement

The authors declare that the research was conducted in the absence of any commercial or financial relationships that could be construed as a potential conflict of interest.

## Supplementary material

Supplementary Figure 1 **Diagrams of rat brain sections showing representative microinjection sites (filled circles) in the NAcC**. Only data from test pairs in which both animals had bilateral needle tracks terminating in the NAcC and no damage to the target tissues were included in the final analyses.
