# Supplementary figures and images for: Interacting Cannabinoid and Opioid Receptors in the Nucleus Accumbens Core Control Adolescent Social Play
Source: Front Behav Neurosci. 2016 Nov 16;10:211. doi: 10.3389/fnbeh.2016.00211 (PMC5110529; doi:10.3389/fnbeh.2016.00211)

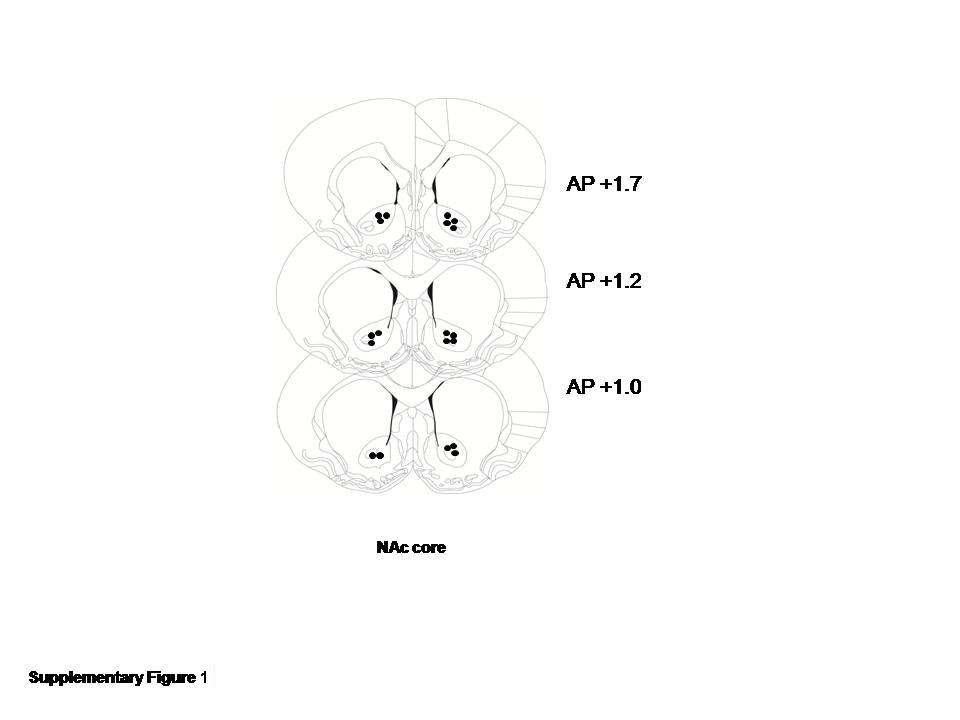

Supplement: Supplementary Figure 1 — Diagrams of rat brain sections showing representative microinjection sites (filled circles) in the NAcC. Only data from test pairs in which both animals had bilateral needle tracks terminating in the NAcC and no damage to the target tissues were included in the final analyses. [file Image1.jpg]
